# Subwavelength core/shell cylindrical nanostructures for novel plasmonic and metamaterial devices

**DOI:** 10.1186/s40580-017-0128-8

**Published:** 2017-12-11

**Authors:** Kyoung-Ho Kim, You-Shin No

**Affiliations:** 1 Department of Chemistry, University of North Carolina at Chapel Hill, Chapel Hill, NC 27599-3290 USA; 2 Department of Physics, Konkuk University, Seoul, 05029 Republic of Korea

**Keywords:** Plasmonics, Metamaterials, Subwavelength nanostructure, Core–shell nanowire

## Abstract

In this review, we introduce novel plasmonic and metamaterial devices based on one-dimensional subwavelength nanostructures with cylindrical symmetry. Individual single devices with semiconductor/metal core/shell or dielectric/metal core/multi-shell structures experience strong light–matter interaction and yield unique optical properties with a variety of functions, e.g., invisibility cloaking, super-scattering/super-absorption, enhanced luminescence and nonlinear optical activities, and deep subwavelength-scale optical waveguiding. We describe the rational design of core/shell cylindrical nanostructures and the proper choice of appropriate constituent materials, which allow the efficient manipulation of electromagnetic waves and help to overcome the limitations of conventional homogeneous nanostructures. The recent developments of bottom-up synthesis combined with the top-down fabrication technologies for the practical applications and the experimental realizations of 1D subwavelength core/shell nanostructure devices are briefly discussed.

## Introduction

One-dimensional (1D) dielectric nanostructures with high refractive indices offer unique opportunities for exploring light-sensitive responses of materials, affording a series of optical resonances that further boost light–matter interaction compared to their bulk counterparts [1–8]. For example, high-refractive-index semiconductor nanowires (NWs) and NW array devices have become attractive platforms for next-generation solar cells since they support strong optical resonances that facilitate the increase of energy conversion efficiency [9–17]. The high quality resonant modes oscillating along the 1D structure provide strongly directional optical properties that are essential in some key applications such as coherent/incoherent optical light sources, requiring a highly directional emission with minimum spatial divergence [1, 2]. Individual subwavelength Si nanostructures (e.g., NWs and nanoparticles) exhibit strong morphology-dependent resonant scattering, allowing their arrays to be used for controllable structural coloration [5, 18]. The excellent optical responses of 1D nanostructure have attracted the increased interest of researchers working in the fields of plasmonics and metamaterials [19–39]. Importantly, plasmonic materials and metamaterials/metasurfaces allow one to overcome the limitations of conventional uniform and homogeneous dielectric materials. One can utilize deep subwavelength-scale optical constituents to expand the functionality of such 1D nanostructure and realize a variety of nanophotonic devices requiring both efficient light manipulation and strong light–matter interaction. In particular, structures with cylindrical symmetry can be readily combined with properly designed metallic components and serve as key building blocks for unique photonic and optoelectronic nanodevices with unprecedented optical functions. For example, the geometrical advantage allows for the 1D core/shell structures to be not only configured into single devices but also easily integrated with other optical and electrical components without breaking the symmetry and distorting the original optical properties, which are hard to obtain from the spherical core/shell nanostructures. In addition, the ability of plasmonic material NWs to support strongly localized surface plasmon modes has led to the development of deep subwavelength plasmonic waveguides [19, 40, 41], optical antennas [20], and surface-enhanced Raman scattering sensors [22, 42, 43]. Rationally designed complex dielectric/metal core/shell or core/multi-shell structures can significantly enhance or suppress the optical responses of nanostructures and allow conventionally inaccessible functionalities such as invisibility cloaking [24–32], super-scattering/super-absorption [33, 34], enhanced luminescence and nonlinear optical activities [35–37], and deep subwavelength optical waveguiding [38, 39]. In this review, we introduce various novel plasmonic and metamaterial devices based on 1D subwavelength core/shell nanostructures. We discuss the rational design of core/shell NW structures composed of materials appropriately chosen for targeted functionalities and successful experimental applications using core/shell NW structures. Moreover, we briefly describe the recently developed synthesis/fabrication technologies for realizing 1D subwavelength core/shell nanowires and their practical applications.

## Review

### Invisible core/shell plasmonic NWs

#### Plasmonic cloaking based on core/shell NW structures

**Fig. 1:**
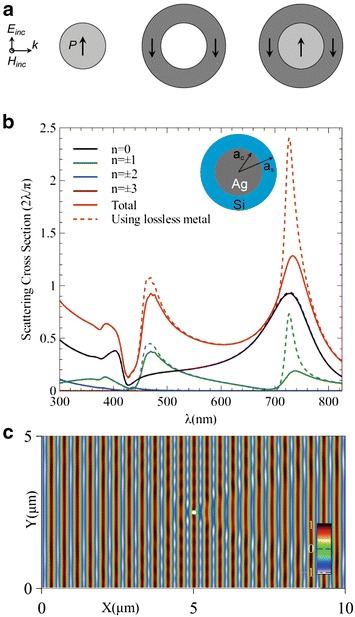
Invisibility cloaking of a metal/semiconductor core/shell plasmonic NW. **a** Qualitative description of plasmonic cloaking in the quasi-static approximation. In a rationally designed core/shell NW, the local polarization vectors of the shell and the core can be anti-parallel (left and middle) and thus cancel each other (right). **b** Total scattering cross-section spectrum (red solid line) of a core/shell NW (Ag core radius *a*_*c*_ = 18 nm and Si shell radius *a*_*s*_ = 73 nm) for TE-polarized light and scattering contributions of individual angular modes. The invisible wavelength was determined at the wavelength of 427 nm where the total scattering cross-section shows a dip and the scattering contribution from each angular mode is minimized. **c** Near-field distribution around the core/shell NW (denoted by a white circle) at the invisible wavelength of 427 nm (**a** is adapted from [24], and **b**, **c** are adapted from [31])

Plasmonic cloaking (proposed by Alù et al.) is based on scattering cancellation, which is a popular method of achieving invisibility [24–27]. One can achieve an efficient scattering cancellation by using spherical or cylindrical core/shell structures forming anti-parallel local polarization vectors in core and shell with a systematically designed cloaking shell material and geometry (Fig. 1a) [24]. The scattering cancellation employs a scheme conceptually different from other cloaking methods, permitting light–matter interaction in the core region [44–48]. It facilitates detecting incident electromagnetic signals without scattering, which can find applications in invisible sensors or photodetectors [29, 49, 50]. Therefore, one can appropriately control the individual polarizations of the core and the shell to develop an interesting plasmonic cloaking system that enables the wavefronts of incident light in the near- and far-field to be preserved without severe distortion.

The essential plasmonic cloaking requirements for core/shell NW structures were first theoretically described by the same research group [25, 27]. For a core/shell NW comprising a homogeneous core and shell with permittivities and permeabilities of (*ε*_*c*_, *μ*_*c*_) and (*ε*_*s*_, *μ*_*s*_), respectively, and having a core radius of *a*_*c*_ and a shell thickness of *a*_*s*_ − *a*_*c*_, the total electric and magnetic fields are expressed by the superposition of incident and scattered light in free space [25]:

1 $$ \begin{aligned} \varvec{E}_{total} = \hat{z}E_{0} \sum\limits_{n = - \infty }^{\infty } {\left[ {i^{n} J_{n} \left( {k_{0} r} \right) + c_{n}^{TM} H_{n}^{\left( 1 \right)} \left( {k_{0} r} \right)} \right]} \, e^{in\phi } \hfill \\ \varvec{H}_{total} = \hat{z}H_{0} \sum\limits_{n = - \infty }^{\infty } {\left[ {i^{n} J_{n} \left( {k_{0} r} \right) + c_{n}^{TE} H_{n}^{\left( 1 \right)} \left( {k_{0} r} \right)} \right]} \, e^{in\phi } \hfill \\ \end{aligned} $$

Equation (1) holds for normal incident light with transverse magnetic (TM; electric field along the NW axis; magnetic field perpendicular to the NW axis) and transverse electric (TE; magnetic field along the NW axis; electric field perpendicular to the NW axis) polarizations, respectively. Here, *J*_*n*_ and *H*_*n*_^(1)^ are the *n*th order cylindrical Bessel and first kind Hankel function, respectively. The *k*_0_ is the wavenumber in free space, and *c*_*n*_^*TM*^ and *c*_*n*_^*TE*^ are the scattering coefficients for TM and TE polarizations, respectively. The NW axis is aligned along the *z*-direction, and the time harmonic term is *e* ^−*iωt*^. The scattering coefficients *c*_*n*_^*TE*^ and *c*_*n*_^*TM*^ can be determined by imposing boundary conditions on the core/shell interface and the NW surface [25, 27]. With these scattering coefficients, the scattering cross-sections for TM and TE polarization are obtained as

2 $$ \begin{aligned} C_{sca}^{TM} = \frac{2\lambda }{\pi }\sum\limits_{n = - \infty }^{\infty } {|c_{n}^{TM} |^{2} } \hfill \\ C_{sca}^{TE} = \frac{2\lambda }{\pi }\sum\limits_{n = - \infty }^{\infty } {|c_{n}^{TE} |^{2} } \hfill \\ \end{aligned} $$

where *λ* is the wavelength in free space [25]. To achieve significant scattering suppression for invisibility cloaking, the scattering coefficients should equal zero. In the subwavelength limit, the conditions for zero *n*th order scattering can be expressed as

3 $$ \begin{aligned} c_{0}^{TM} :\frac{{a_{s} }}{{a_{c} }} = \sqrt {\frac{{\varepsilon_{s} - \varepsilon_{c} }}{{\varepsilon_{s} - \varepsilon_{0} }}} ,\quad c_{n \ne 0}^{TM} :\frac{{a_{s} }}{{a_{c} }} = \sqrt[{2n}]{{\frac{{\left( {\mu_{s} - \mu_{c} } \right)\left( {\mu_{s} + \mu_{0} } \right)}}{{\left( {\mu_{s} - \mu_{0} } \right)\left( {\mu_{s} + \mu_{c} } \right)}}}}, \hfill \\ c_{0}^{TM} :\frac{{a_{s} }}{{a_{c} }} = \sqrt {\frac{{\mu_{s} - \mu_{c} }}{{\mu_{s} - \mu_{0} }}} ,\quad c_{n \ne 0}^{TM} :\frac{{a_{s} }}{{a_{c} }} = \sqrt[{2n}]{{\frac{{\left( {\varepsilon_{s} - \varepsilon_{c} } \right)\left( {\varepsilon_{s} + \varepsilon_{0} } \right)}}{{\left( {\varepsilon_{s} - \varepsilon_{0} } \right)\left( {\varepsilon_{s} + \varepsilon_{c} } \right)}}}} \, \hfill \\ \end{aligned} $$

where *ε*_0_ and *μ*_0_ are the permittivity and permeability in free space, respectively [25, 27]. Thus, the total net scattering from NWs can be effectively suppressed when the Eq. (3) is satisfied. Ali et al. explored these critical conditions and proposed silver core/silicon shell NWs with carefully determined geometrical parameters (inset of Fig. 1b) [31]. They successfully demonstrated a considerable reduction of the total scattering cross-section at the target wavelength (called the invisible wavelength) in the visible frequency range (Fig. 1b). To explain the observed suppression, the total scattering was decomposed into individual *n*th angular modes and their scattering contributions in the proposed structure were shown to be minimized. In addition, the calculated near-field distribution around the proposed NWs at the invisible wavelength clearly visualized the weak interference patterns and confirmed the validity of the cloaking mechanism (Fig. 1c).

#### Invisibility cloaking of a hybrid metal–semiconductor NW structure

**Fig. 2:**
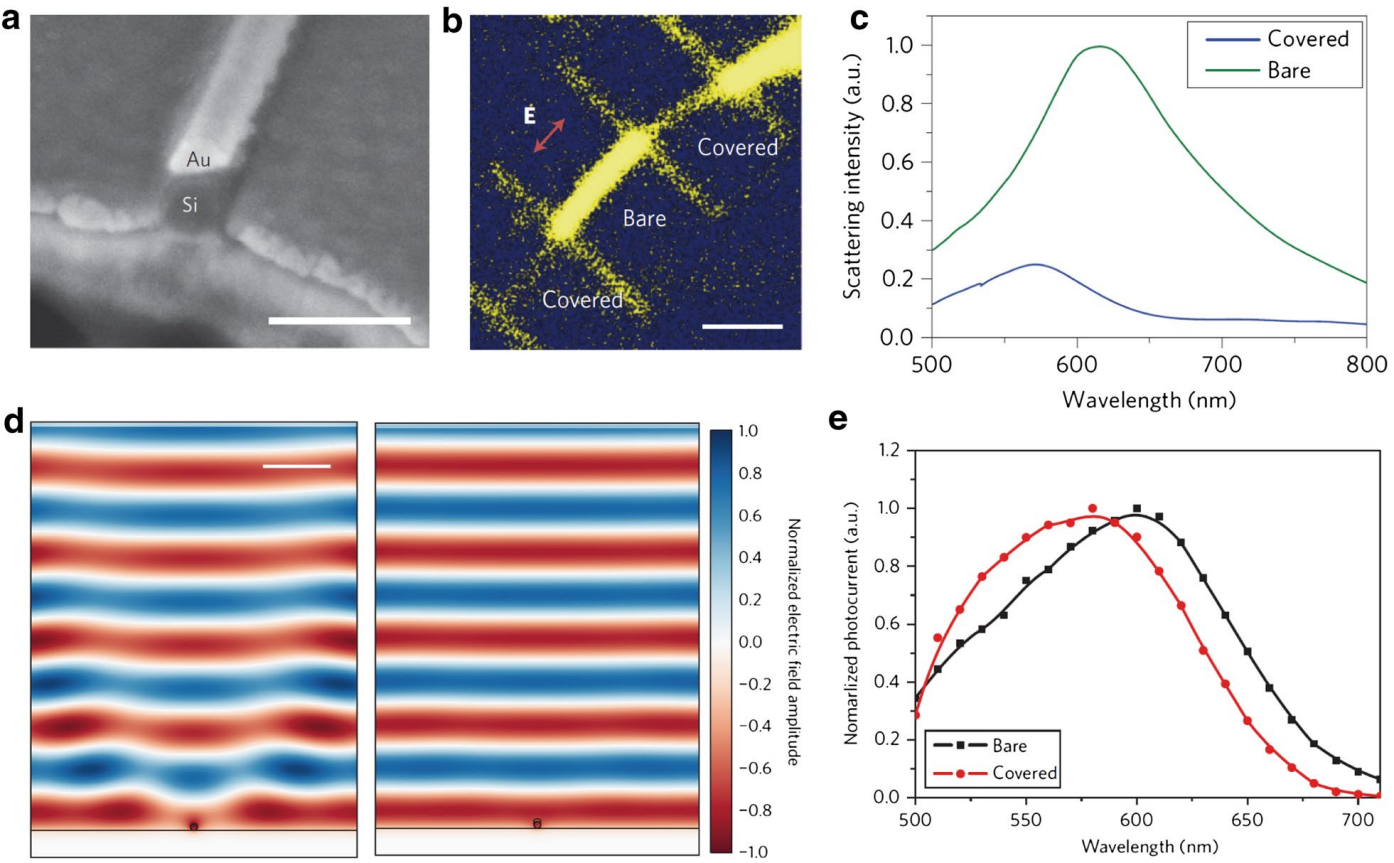
Experimental demonstration of plasmonic cloaking and an invisible NW photodetector. **a** Scanning electron microscopy (SEM) image of a plasmonic NW device comprising a 50-nm-diameter Si NW and a 20-nm-thick gold cover. Scale bar, 100 nm. **b** Confocal optical microscopy image of the device in **a** under TM-polarized incident light illumination, with gold-coated and uncoated areas indicated as “covered” and “bare”, respectively. Scale bar, 2 μm. **c** Measured scattering intensity spectra from bare (green) and covered (blue) segments of the Si NW under TM-polarized light illumination. **d** Simulated near-field distributions around Si NW with (right) and without (left) the gold cover under the top-illuminated TM-polarized incident light at wavelength of 650 nm. Scale bar, 500 nm. **e** Measured spectral photocurrents of the 50-nm-diameter Si NW with (red) and without (black) the 20-nm-thick gold cover (**a**–**e** are adapted from [29])

Invisibility by plasmonic cloaking has been experimentally demonstrated for subwavelength-scale semiconductor NWs and novel metal covers (Fig. 2) [29]. Researchers at Stanford University fabricated a hybrid metal–semiconductor plasmonic NW device comprising a single Si NW with a diameter of 50 nm and a gold cover with a thickness of 20 nm (Fig. 2a). They used confocal microscopy to measure the intensity of light backscattered from gold-coated and uncoated segments (Fig. 2b) and revealed that the “covered” NW segment exhibited dramatically suppressed light scattering compared to the “bare” segment of the same NW. Moreover, the broadband suppression of scattering from the “covered” segment in the visible spectrum made the NW essentially invisible (Fig. 2c).

The demonstrated invisibility of the gold-covered Si NW was explained by scattering cancellation [24]: the Si NW and the judiciously designed gold cover were oppositely polarized with an equal magnitude, affording a net-zero local polarization vector and thus resulting in scattering cancellation. The near-field distribution around the Si NW obtained by full-wave numerical simulations further supported the experimentally observed plasmonic cloaking (Fig. 2d). Without the gold cover, the subwavelength-scale bare Si NW distorted the regular interference pattern (left, Fig. 2d), whereas the presence of the gold cover restored the planar wavefronts and made the NW invisible (right, Fig. 2d). Moreover, the authors have demonstrated that the gold covered Si NW can be an invisible photodetector. They measured spectral photocurrents for gold-coated and uncoated regions revealed that the photocurrent at the invisible wavelength of ~ 650 nm was only decreased by a factor of four, whereas plasmonic cloaking suppressed scattering by over two orders of magnitude. These results indicate that the described plasmonic NW device can be utilized as an invisible sensor for the detection of optical signals without near- and far-field distortion [49, 50].

#### Subwavelength metasurface and metamaterial shell structures

**Fig. 3:**
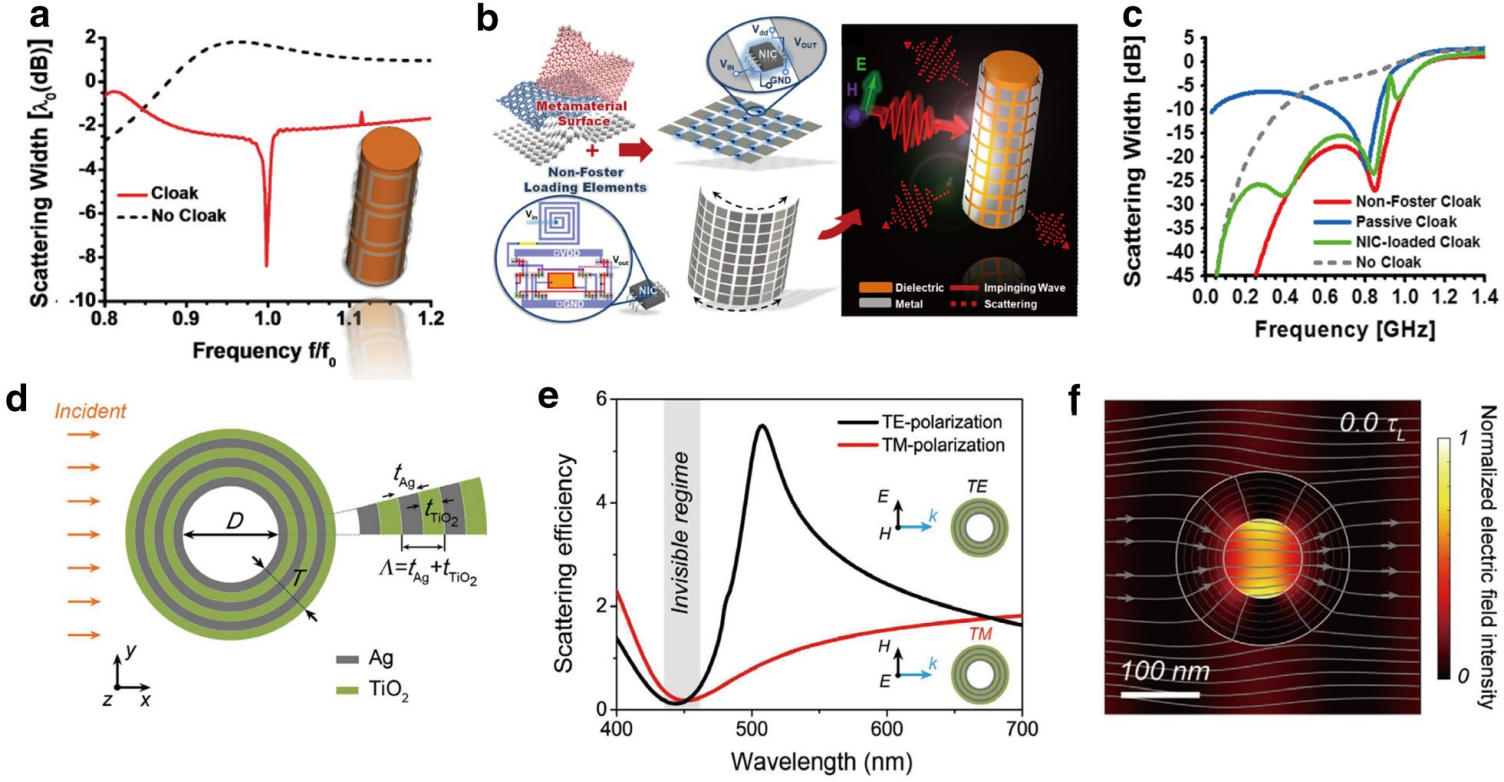
Subwavelength cylindrical metasurface and metamaterial shell structures for invisibility cloaking. **a** Scattering width spectra (TM-polarized light) of an infinitely long dielectric cylinder with (red solid line) and without (black dashed line) a metasurface mantle cloak. Inset: schematic illustration of the metasurface mantle cloaked device. The dielectric core has an electric permittivity of 10 and the diameter of *λ*/4, where *λ* is the invisibility wavelength. The metasurface comprises an array of square metallic rings with a thickness of *λ*/400. **b** Concept of a broadband metasurface mantle cloak on an infinite dielectric cylinder featuring an array of ultrathin curved metal patches loaded with non-Foster elements. **c** Normalized scattering width spectra of a dielectric core covered with various metasurface mantle cloaks. **d** Schematic illustration of a coaxial metal/dielectric multilayered metamaterial nanotube. The nanotube has an air core (diameter = *D*) and a metamaterial shell (thickness = *T*) comprising multiple layers of alternating Ag/TiO_2_ films. **e** Scattering efficiency spectra of a layered nanotube structure with a core diameter of 100 nm and a shell thickness of 60 nm for TE- (black line, top inset) and TM- (red line, bottom inset) polarized light. The thicknesses of both individual Ag and TiO_2_ layers are set to 10 nm. The invisible region is indicated by a shaded gray area. **f** Snapshot of the normalized electric field intensity distribution and time-averaged Poynting vector power flows (gray lines and arrows) near the metal/dielectric layered nanotube at the invisible wavelength of ~ 450 nm (**a** is adapted from [28], **b**, **c** are adapted from [30], and **d**–**f** are adapted from [32])

Light scattering can be suppressed not only by using a homogeneous plasmonic shell but also by employing complex patterned surfaces or shell structures such as metasurfaces and metamaterials [28, 30, 32]. Recently, an intriguing concept of metasurface mantle cloaking was proposed by Chen et al. [28]. They elaborately designed various ultrathin conducting metasurfaces to cover dielectric objects with different shapes (i.e., planar slabs, infinite cylinders, and spheres) and achieved a dramatic reduction of light scattering in the radio and terahertz frequency range. The basic mechanism of mantle cloaking is similar to that of plasmonic cloaking, i.e., the metasurface produces an anti-phased scattered wave and cancels most of the scattered fields, effectively making the object invisible [24, 27]. For example, Fig. 3a shows that a properly designed metasurface can considerably reduce the scattering width from a dielectric cylinder at the target cloaking wavelength [28]. However, such drastic suppression of scattering from a given dielectric object implies that the operation bandwidth is confined within a certain range due to the resonance and dispersive nature of inclusions in metasurfaces, which prevents this device concept to be further extended to a broader frequency range. To overcome these limitations, researchers have improved the mantle cloaking concept and proposed a modified metasurface (termed a non-Foster metasurface) [30]. The proposed metasurface includes active elements [e.g., negative impedance converters (NICs)] for which Foster’s theorem does not apply. Notably, the active elements release the inherent dispersive nature of optical response of the metasurface and thus allow the cloaking bandwidth to be extended (Fig. 3b). Figure 3c shows the calculated scattering width spectra of an infinitely long dielectric cylinder with various types of mantle cloaks, revealing that NIC-loaded (green solid line) and non-Foster (red solid line) mantle cloaks exhibit significantly improved cloaking bandwidth compared to that of the passive metasurface mantle cloak (blue solid line).

The increased attention enjoyed by invisibility cloaking of subwavelength-scale objects partly comes from the availability of suitable layered metamaterials [32]. In particular, the concept of hyperbolicity in momentum space has been successfully utilized to explore the invisibility of single nanostructures in the visible frequency range. Kim et al. proposed a radial anisotropic hyperbolic metamaterial nanotube comprising an air core and a layered shell structure featuring multiple alternating thin layers of Ag and TiO_2_ (Fig. 3d) and numerically investigated its scattering properties under both TE- and TM-polarized incident light [32]. Since the permittivities of Ag and TiO_2_ exhibit opposite signs in the visible frequency range, the optical response of the nanotube can be effectively tuned by controlling the thickness of constituent layers. Figure 3e shows the calculated scattering efficiency spectra of the metamaterial nanotube, revealing that scattering is dramatically reduced at a wavelength of ~ 450 nm under TE- and TM-polarized incident light, consequently leading to decreased visibility. To gain further insights, the authors modeled the layered nanotube as a radially anisotropic hyperbolic metamaterial nanotube [51, 52]. They showed that the decreased scattering occurs when the effective permittivity of the hyperbolic nanotube in the angular direction was close to zero, which is distinguishable from that of conventional plasmonic cloaking. Furthermore, the near-field intensity distribution at the invisible wavelength revealed the occurrence of unique field enhancement inside the nanotube (Fig. 3f), which was explained by the focusing of time-averaged Poynting vector power flow in the core region with a volume of less than ~ 0.16 (*λ*/2*n*)^2^, where *λ* is the wavelength of incident light and *n* is the refractive index of the core. Such strong light focusing is expected to be useful for various nanophotonic applications requiring strong light–matter interaction for invisible sensors as well as for spontaneous emission enhancement for fluorescence measurements without signal distortion by scatterers [53, 54].

### Super-scattering and super-absorption

The invisibility with suppressed scattering is not only the achievable optical feature of layered subwavelength 1D nanostructures. The enhanced scattering and strong absorption beyond the fundamental limits are also attainable using the core/shell 1D nanostructures. Light scattering and absorption are caused by the interaction of light with individual nanostructures. The strength of the interaction is often determined by physical dimension of the nanostructure and the supported optical resonances as well as the material compositions [55]. For example, the total scattering (*C*_sca_) and absorption (*C*_abs_) cross-sections of a subwavelength homogeneous wire can be expressed as

4 $$ \begin{aligned} C_{\text{sca}} = \sum\limits_{n = - \infty }^{\infty } {C_{{{\text{sca}},n}} } \hfill \\ C_{\text{abs}} = \sum\limits_{n = - \infty }^{\infty } {C_{{{\text{abs}},n}} } \hfill \\ \end{aligned} $$

**Fig. 4:**
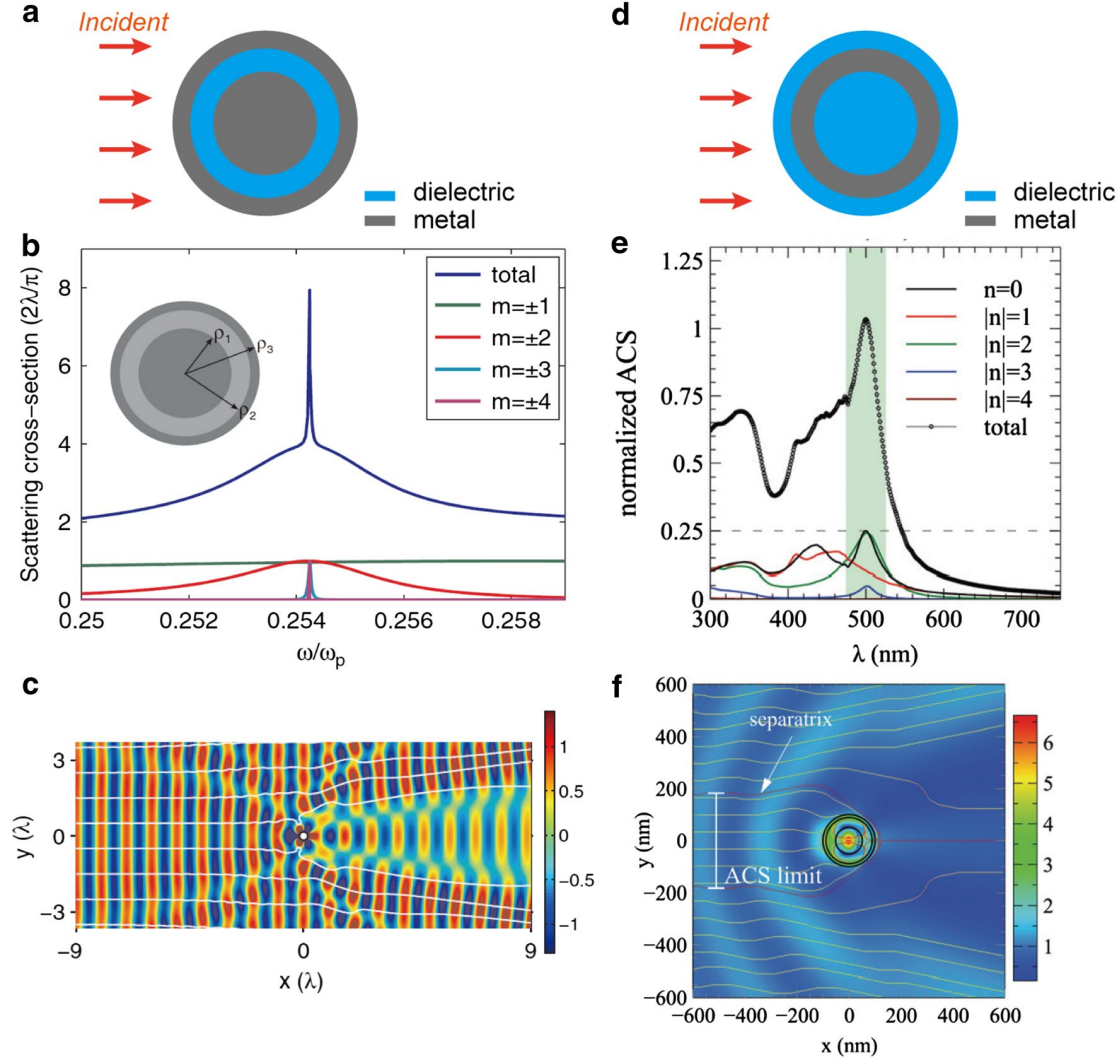
Super-scattering and super-absorption of multilayered coaxial metal/dielectric nanostructures. **a** Schematic illustration of a core/multi-shell NW structure for super-scattering. The NW is composed of a metal core and a layered dielectric/metal multi-shell. **b** Scattering cross-section spectra of the NW in the lossless case. Total scattering and scattering contributions of individual angular modes are shown. The permittivity of the dielectric layer was set to 12.96. The permittivity of the metal layer was described by the Drude model with *ε* = 1 − *ω*_p_^2^/*ω* ^2^ where ω_p_ is the plasma frequency (lossless case). The inset shows the geometrical parameters of the NW: *ρ*_1_ = 0.3485*λ*_p_, *ρ*_2_ = 0.5623*λ*_p_, and *ρ*_3_ = 0.6370*λ*_p_, where *λ*_p_ = 2π*c*/*ω*_p_ and *c* is the speed of light in vacuum. **c** Near-field distribution and Poynting vector lines of the super-scattering NW at a frequency of 0.2542*ω*_p_. **d** Schematic illustration of a core/multi-shell NW for super-absorption. The NW is composed of a dielectric core and a layered metal/dielectric multi-shell structure. **e** Normalized absorption cross-section spectra of the NW. Total absorption and absorption contributions of individual angular modes are shown. Si and Ag were used as dielectric and metallic materials, respectively. The geometrical parameters, *ρ*_1_, *ρ*_2_ and *ρ*_3_ for the NW were set to 51, 92 and 100 nm, respectively. **f** Near-field distribution and Poynting vector lines of the super-absorption NW at a wavelength of ~ 500 nm (**b**, **c** are adapted from [33], and **e**, **f** are adapted from [34])

In the presence of a resonance in the *n*th angular momentum channel, *C*_sca,*n*_ and *C*_abs,*n*_ become

5 $$ \begin{aligned} C_{{{\text{sca}},n}} = \frac{2\lambda }{\pi }\frac{{\gamma^{2} }}{{\left( {\omega - \omega_{0} } \right)^{2} + \left( {\gamma_{0} + \gamma } \right)^{2} }} \hfill \\ C_{{{\text{abs}},n}} = \frac{2\lambda }{\pi }\frac{{\gamma \gamma_{0} }}{{\left( {\omega - \omega_{0} } \right)^{2} + \left( {\gamma_{0} + \gamma } \right)^{2} }} \hfill \\ \end{aligned} $$

where $\omega$ is the frequency of incident wave, $\omega_{0}$ is the resonant frequency, $\gamma_{0}$ is the intrinsic loss rate due to material absorption, and $\gamma$ is the external leakage rate caused by the coupling of the resonance to the outgoing wave [33, 34]. Therefore, in the strong coupling limit, i.e., $\gamma \gg \gamma_{0}$, the maximum scattering cross-section of a single angular momentum channel is in principle limited by 2*λ*/*π*. Moreover, at the critical coupling condition, $\gamma = \gamma_{0}$, the absorption cross-section is maximized to *λ*/2*π*. However, the recently proposed concepts of super-scattering and super-absorption utilize multilayered core/shell NWs to overcome these conventional limitations (Fig. 4) [33, 34]. The proposed strategy is to create multiple resonances from various angular momentum channels at the same frequency and ensures that they are operated in either the strong coupling limit (super-scattering) or the critical coupling limit (super-absorption). For example, Ruan et al. designed a subwavelength super-scattering NW by combining a plasmonic core with a layered dielectric/plasmonic shell (Fig. 4a) [33]. Figure 4b exhibits the aligned resonances from individual channels and the strongly enhanced total scattering cross-section. The near-field distribution around the NW directly visualizes the strong interference pattern caused by super-scattering, as shown in Fig. 4c. For super-absorption, Mirzaei et al. proposed a similar core/shell structure with an opposite material composition to control the resonant absorption from individual channels and align them in a single frequency (Fig. 4d) [34]. Figure 4e shows that successfully tuned resonant absorptions from individual channels strongly enhance the total absorption cross-section at the target wavelength of ~ 500 nm. The calculated field distribution and energy flow lines close to the NW clearly revealed that incident light was trapped and absorbed in the semiconducting material (Fig. 4f). Taken together, the use of subwavelength NWs comprising concentric metal/semiconductor multilayers allows the realization of unusually strong light–matter interactions and enables enhanced light scattering and absorption.

### Plasmonic cavities for enhanced luminescence and second harmonic generation (SHG)

**Fig. 5:**
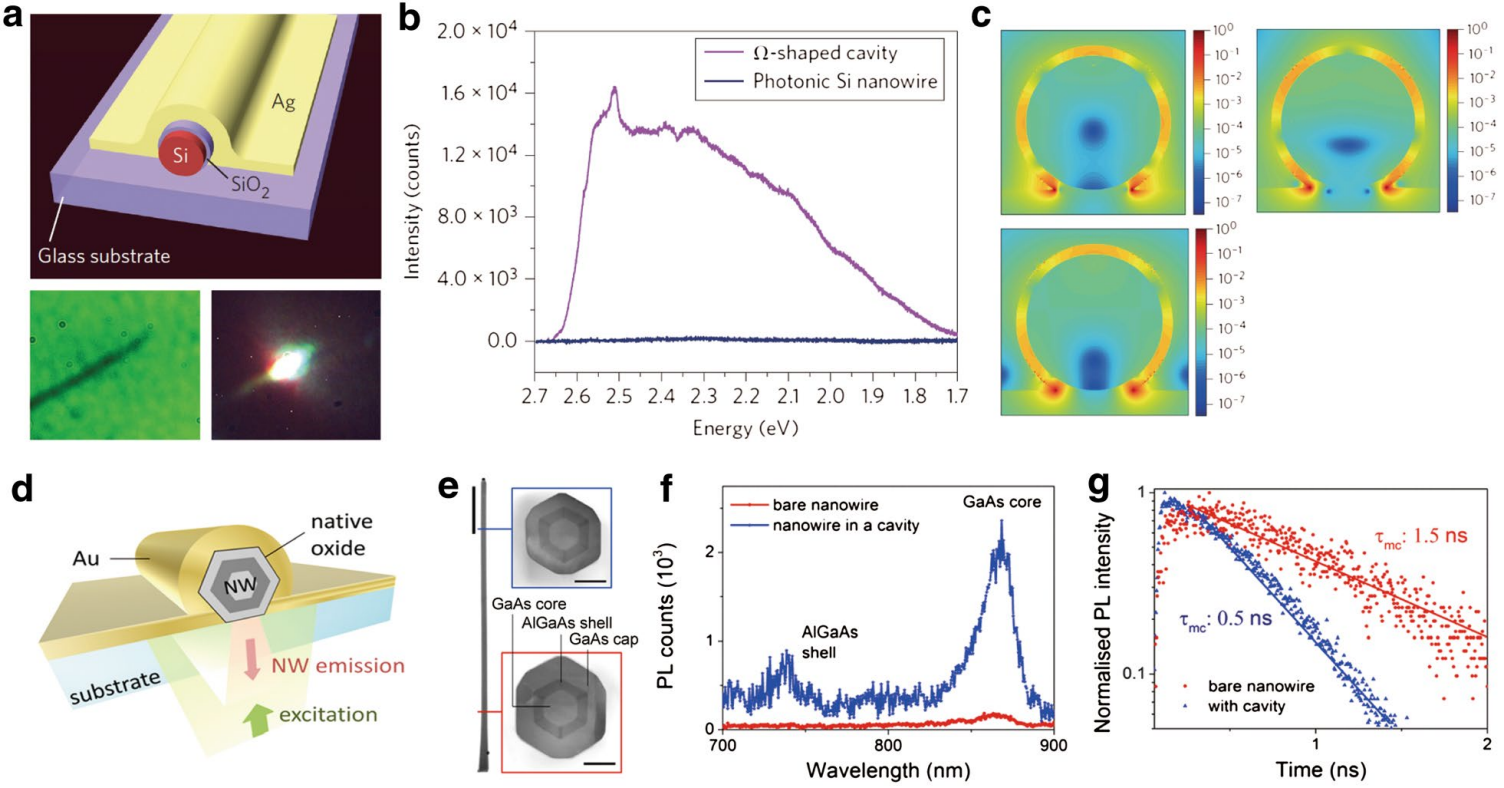
Strong enhancement of luminescence and quantum efficiency of NWs coupled to plasmonic nanocavities. **a** Schematic illustration of a Si NW coupled to an Ω-shaped Ag plasmonic nanocavity device for visible hot luminescence (top). Optical microscopy images of the fabricated single plasmonic NW device (bottom, left) and its hot luminescence recorded under focused laser excitation (bottom, right). **b** Photoluminescence spectra of a bare Si NW (blue) and a Si NW coupled to an Ω-shaped plasmonic nanocavity (magenta) at room temperature. **c** Calculated electric field intensity profiles of various plasmonic modes excited in the NW plasmonic nanocavity. The diameter of Si NW is 70 nm. **d** Schematic illustration of a III–V NW coupled to an Ω-shaped Au plasmonic nanocavity to achieve high quantum efficiency. **e** SEM images of a core/shell/cap GaAs/AlGaAs/GaAs NW showing diameter variations along the NW length. **f** Measured photoluminescence spectra of a bare GaAs/AlGaAs/GaAs core/shell/cap NW (red) and a NW coupled to an Ω-shaped plasmonic nanocavity (blue). **g** Time-resolved photoluminescence data obtained from the bare and nanocavity-coupled NWs. The estimated minority carrier lifetimes are 1.5 and 0.5 ns for the bare and nanocavity-coupled NWs, respectively (**a**–**c** are adapted from [35] and **d**–**g** are adapted from [36])

Owing to their strong light–matter interaction, cylindrical plasmonic core/shell nanostructures are useful for increasing the luminescence efficiency of semiconductor NWs. For example, light emission from Si-based sources has been a major challenge due to their extremely low emission efficiency originating from the indirect bandgap of Si [56]. Cho et al. utilized the core/shell plasmonic nanocavities and achieved bright visible light emission in Si NWs (Fig. 5a) [35]. The plasmonic NW device consists of a Si NW (red) covered with thin layers of SiO_2_ (purple) and Ag (yellow) on a glass substrate, which forms an Ω-shaped plasmonic nanocavity. Direct comparison of measured photoluminescence spectra of bare and nanocavity-coupled Si NWs clearly shows a significant increase of light emission in the visible spectral range in the latter case (Fig. 5b). Using highly concentrated electromagnetic fields inside plasmonic nanocavities (Fig. 5c), they successfully realized light emission from hot carriers with high quantum yield (> 1%) at room temperature.

The NW/metal core/shell plasmonic nanocavity structural concept can also be used to enhance the emission quantum efficiency of conventional III–V compound semiconductor NWs, which has been challenging even for direct band gap semiconductor materials due to their large surface-area-to-volume ratio. This large ratio results in a high density of surface defects [57], which causes the undesirable surface non-radiative recombination and subsequently limits quantum efficiency and NW luminescence. For example, Mokkapati et al. demonstrated that the quantum efficiency of GaAs NWs can be increased by an order of magnitude when Ω-shaped Au nanocavities are used [36]. Additionally, the authors revealed that the increased quantum efficiency originated from the enhanced radiative recombination (Fig. 5d). In this work, the GaAs NW core was passivated with an AlGaAs shell, and an additional GaAs layer was introduced to prevent the oxidation of Al in this shell (Fig. 5e). Next, the obtained core/shell/cap GaAs/AlGaAs/GaAs NWs were coupled to Ω-shaped Au nanocavities. From the measured photoluminescence spectra, they observed ~ 10 times stronger GaAs emission peak from the NW coupled with Ω-shaped Au nanocavity than that from the bare NW (Fig. 5f). Time-resolved photoluminescence measurements of the GaAs NWs at their emission peak also showed that the minority carrier lifetime was reduced from 1.5 ns (bare NW) to 0.5 ns (NW coupled to an Ω-shaped Au nanocavity) (Fig. 5g). The enhanced photoluminescence intensity and the reduced minority carrier lifetime were ascribed to the increased radiative recombination rate in the nanocavity-coupled NW. Based on experimental results, the authors estimated that nanocavity coupling enhanced the quantum efficiency by as much as 900%.

**Fig. 6:**
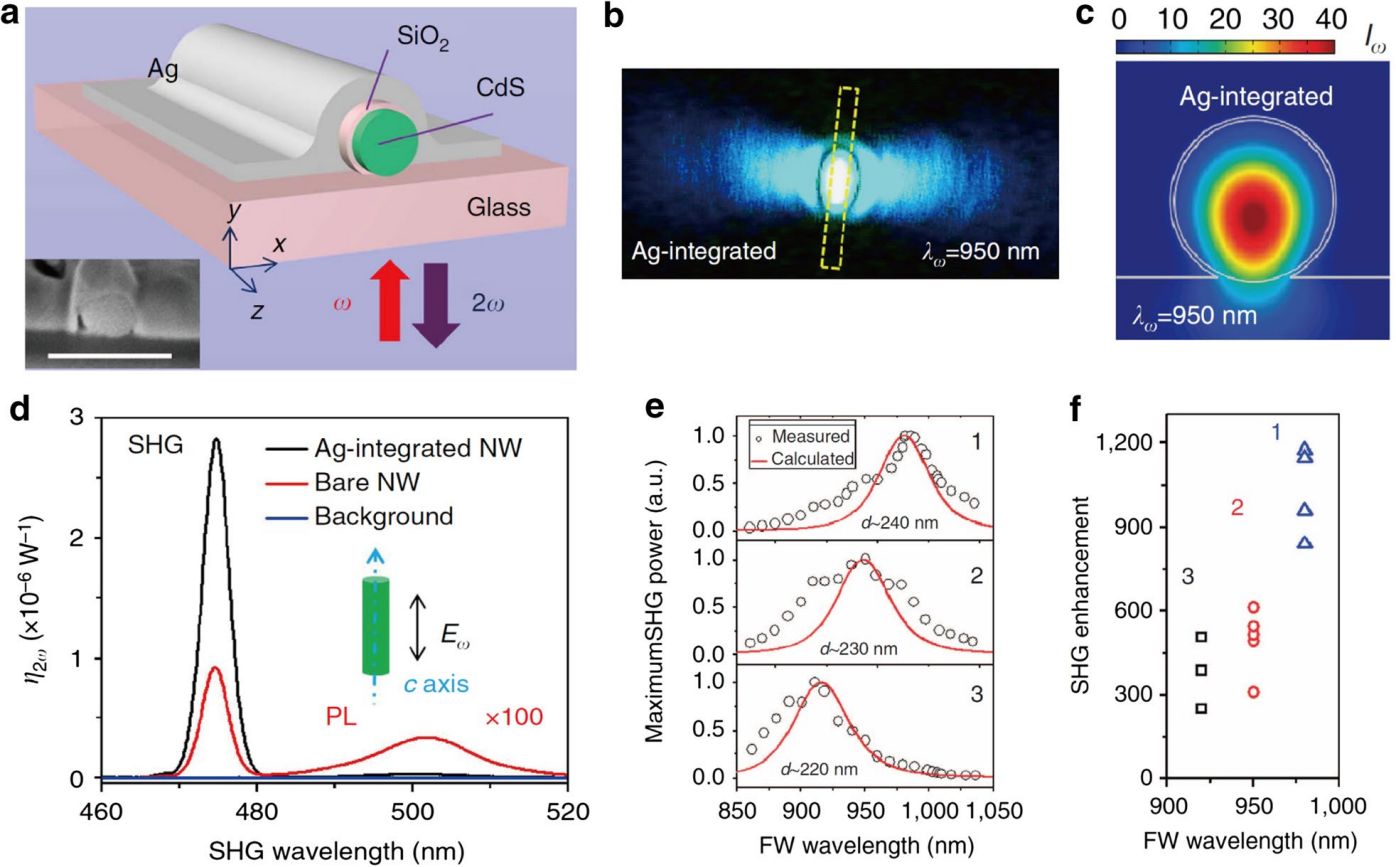
Enhanced second harmonic generation from CdS NWs coupled to plasmonic nanocavities. **a** Schematic illustration of a plasmonic NW device for enhanced SHG comprising a nonlinear CdS NW covered with Ag nanocavity. The fundamental wave (ω) is illuminated through the glass substrate while collecting the back-scattered second harmonic (2*ω*) signals. Inset shows an SEM image of the fabricated plasmonic NW device. **b** Dark-field optical microscopy image showing SHG signals (blue) from a CdS NW coupled to a Ag nanocavity device (yellow dashed rectangle). **c** Calculated electric field intensity distribution (*I*_ω_ = |*E*_ω_|^2^) of the fundamental wave. **d** Estimated SHG conversion efficiencies as a function of SHG wavelength emitted from plasmonic nanocavity-integrated (black) and bare (red, ×100) CdS NWs. **e**, **f** Size- and wavelength-dependent SHG power and enhancement (**a**–**f** are adapted from [37])

Improved optical nonlinearities have also been achieved by integrating nonlinear semiconductor NWs with cylindrical plasmonic core/shell nanocavities. Since the nonlinear optical response strongly depends on the interaction between fields of fundamental wave and nonlinear materials, designing high quality optical cavities has been a key strategy to achieve improved nonlinear activities. However, dielectric optical cavities with high Q-factors result in a large device footprint, which limits applications in ultracompact integrated nanophotonic systems. Conversely, nanoscale nonlinear dielectric-metal structures with a small footprint provide local field enhancement but suffer from poor spatial field overlap and intrinsic ohmic losses at the interface. To address these issues in nonlinear NW optical devices, Ren et al. have employed an Ω-shaped plasmonic nanocavity (Fig. 6a) and demonstrated strongly enhanced SHG from CdS NWs [37]. To excite SHG signals (~ 475 nm), the plasmonic NW device (yellow dashed rectangle in Fig. 6b) was pumped by using a fundamental wave with a wavelength (*λ*_ω_) of 950 nm. The generated SHG light symmetrically radiated from the NW in the lateral direction (Fig. 6b). The Ω-shaped plasmonic nanocavity enables strong concentration of fundamental wave inside the NWs, which increases the field overlap with the nonlinear material without significant ohmic loss caused by the metallic shell (Fig. 6c). Based on measured fundamental wave and SHG peak powers, the introduction of a Ag nanocavity increased the conversion efficiency of the NWs ~ 300-fold (Fig. 6d). Moreover, the power of SHG signals was demonstrated to be NW size-dependent due to the related frequency shift of the resonant mode (Fig. 6e), e.g., a more than 1000-fold enhancement was observed for a nanocavity-coupled CdS NW with a diameter (*d*) of ~ 240 nm (Fig. 6f).

### Graphene-coated NWs for deep-subwavelength-scale graphene plasmon guiding

**Fig. 7:**
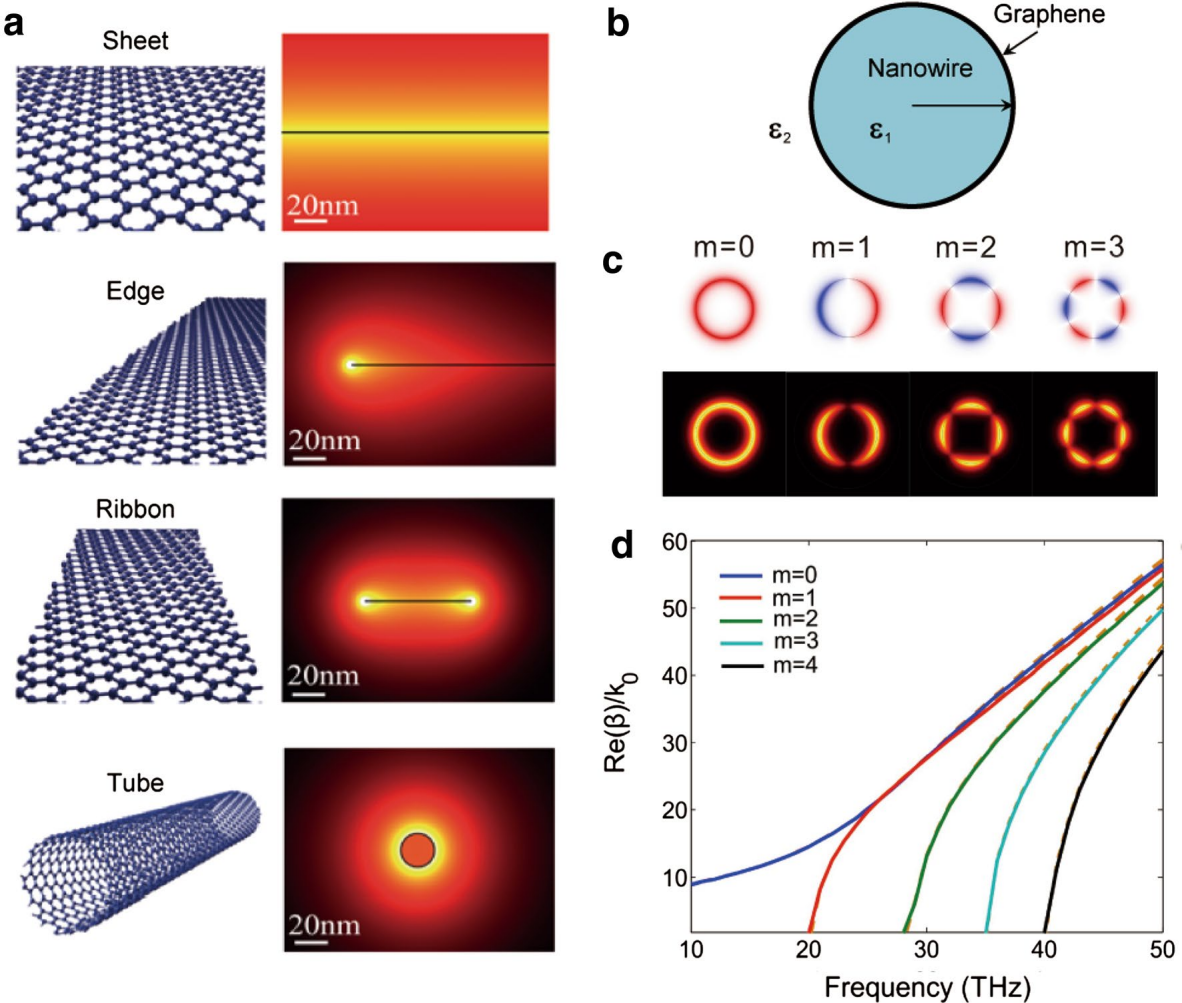
Excitation and guiding of graphene plasmons in various geometrical configurations. **a** Plasmonic modes in different graphene structures: infinite sheet, edge, ribbon and single-walled tube (left), and the corresponding graphene plasmon modes (right). **b** Schematic illustration of a graphene-coated NW. **c** Electric field (top) and intensity profiles (bottom) of various graphene plasmon modes with different angular mode numbers. **d** Dispersions of guided graphene plasmon modes with different angular mode numbers propagating along the graphene-coated NW with a radius of 100 nm. The permittivity of the background and the NW were set to one and three, respectively, and the chemical potential of graphene was set to 0.5 eV (**a** is adapted from [38] and **b**–**d** are adapted from [39])

Optical waveguides, key elements for all-optical signal processing, rely on the strong confinement of electromagnetic energy to efficiently transport it to the desired location. Graphene, an atomically thin two-dimensional carbon material, has drawn considerable attention as an emerging platform for guiding infrared and terahertz frequency waves due to its ability to transport graphene plasmons with ultrashort wavelengths and high field confinement [58, 59]. In addition, graphene plasmons can be controlled by changing the carrier density of graphene via electrostatic gating [60, 61]. So far, a variety of graphene geometries are known to support graphene plasmons, e.g., sheets, edges, nanoribbons, and graphene tubes (Fig. 7a). Among these structures, graphene plasmons supported by graphene-coated NW show distinct waveguiding features compared to other geometries (Fig. 7b) [39]. The NW diameter can be much smaller than the photon wavelength in free space, which is desirable for subwavelength-scale photonic devices and allows the integration density in a photonic circuit to be increased. Moreover, graphene is typically rolled into a cylinder that covers the NW and thus has no edges or discontinuities, which prevents undesired imperfection-induced optical loss. Finally, the azimuthal symmetry of the cylinder allows multiple guided modes with different angular mode distributions (Fig. 7c). The dispersion curves of the guided graphene plasmon modes in Fig. 7d reveal that higher-order plasmon modes with angular mode number *m* > 0 exhibit cut-off frequencies, whereas the fundamental mode exhibits no cut-off frequency, which is desirable for a single-mode waveguide. Furthermore, the high effective mode indices correspond to highly localized graphene plasmons on the graphene/NW interface with a significantly reduced plasmon wavelength compared with the photon wavelength in free space, allowing strong light–matter interaction in infrared and terahertz frequency regime. In conclusion, the described features of graphene plasmons make graphene-coated NWs promising waveguides and key building blocks for integrated tunable plasmonic nanodevices for infrared and terahertz frequencies [59, 62].

### Synthesis of conformal and uniform multilayer core/shell cylindrical micro/NWs for novel plasmonic and metamaterial devices

**Fig. 8:**
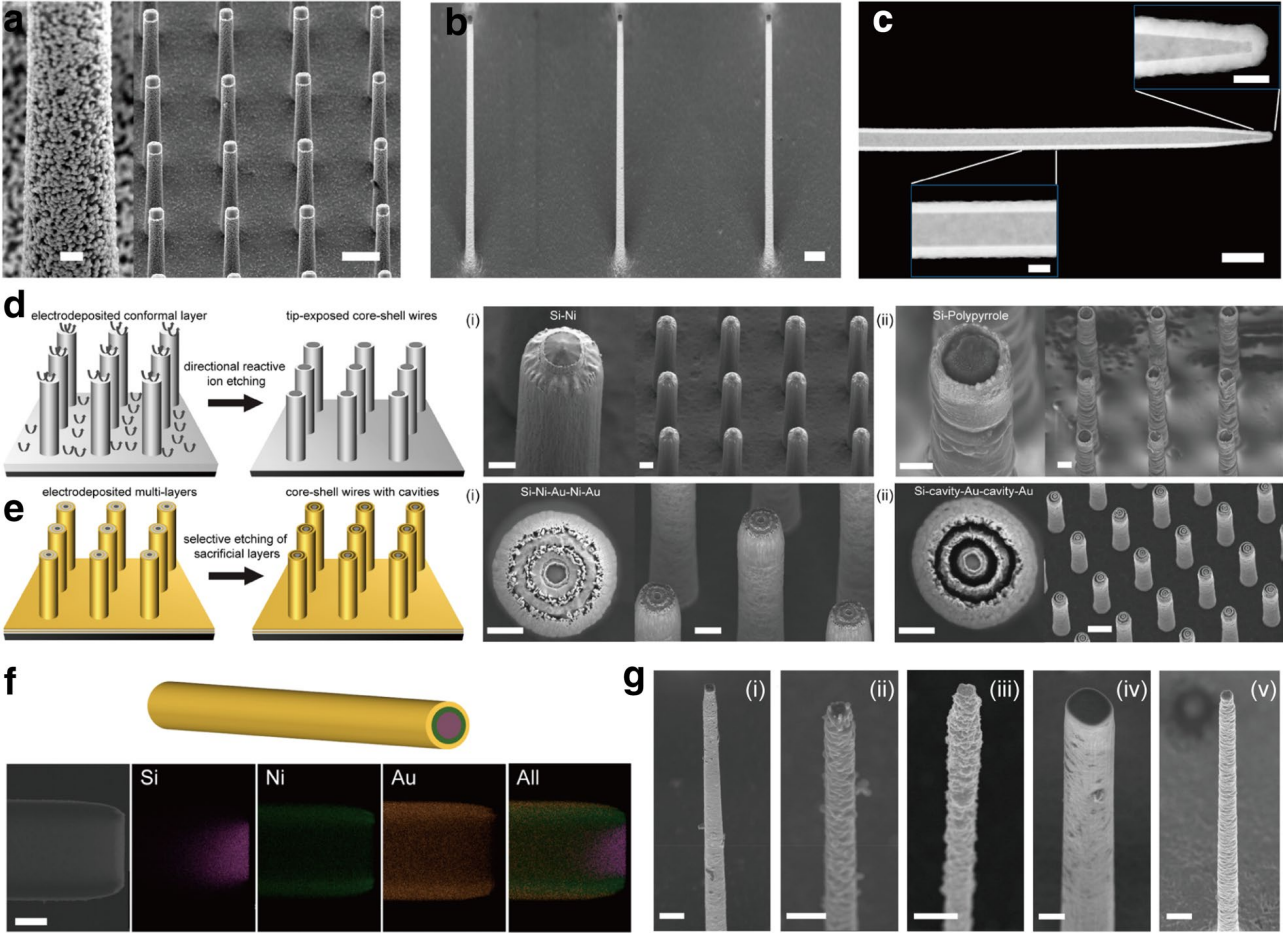
Conformal and uniform deposition of various materials on high-aspect-ratio silicon micro/nanowires for coaxial/multilayered cylindrical architectures. **a** Top-down fabricated Si wire array homogeneously decorated with Ni nanoparticles. **b** High-aspect-ratio Si NWs covered with a thin Ni layer. **c** Low- and high-resolution STEM images for detailed characterization of a uniformly deposited Ni film on a Si wire. **d** Schematic illustration of tip-exposure of core/shell wires (left) and SEM images of tip-exposed core/shell Si/Ni (middle) and Si/polypyrrole (right) wires. **e** Schematic illustration of multilayer uniform deposition for core/shell wires (left) and SEM images of Si/Ni/Au/Ni/Au (middle) and Si/cavity/Au/cavity/Au (right) wires. **f** Schematic illustration of a Si/Ni/Au core/shell NW (top), STEM image of the fabricated wire (bottom, left) and corresponding elemental mapping images of the above wire. **g** Deposition of different materials on Si wires: (i) rhodium, (ii) polyaniline, (iii) cadmium selenide, (iv) Ni/polyaniline shell, (v) Ni/Ru shell (**a**–**g** are adapted from [63])

There has been extensive theoretical studies and detailed numerical characterizations of unique electromagnetic behaviors in various types of cylindrical layered plasmonic and metamaterial nanodevices. However, their experimental demonstrations have been limited by the lack of strategy to realize such cylindrical nanostructures that require precisely controlled synthesis and nanofabrication. In particular, it is difficult to define a conformal and uniform layer of metal on high-aspect-ratio micro/nanoscale wires to form dielectric or semiconductor/metal core/shell structures. Recently, Ozel et al. reported a powerful synthetic method of electrochemical deposition for conformal and uniform layers of various materials on high-aspect-ratio Si micro/nanowires [63]. The authors demonstrated the general applicability of this technique by depositing various metals (Au, Ag, Cu, Pt, Pd, Ru, Rh, Ni, Fe), catalytically active metal oxides (MnO_*x*_, CoO_*x*_), semiconducting metal chalcogenides (CdS, CdSe), and conducting polymers [polyaniline, polypyrrole, poly(3,4-ethylenedioxythiophene)] on 1D high-aspect-ratio Si micro- and nanowires. Selected examples of the above results are shown in Fig. 8. Figure 8a shows top-down fabricated Si wires homogeneously decorated by a layer of Ni nanoparticles. Defining a thin layer of Ni with controlled thickness and uniformity on the Si wires is also demonstrated (Fig. 8b, c). Moreover, the conformal deposition of a thin layer of Ni [(i), Fig. 8d] or polypyrrole [(ii), Fig. 8d] was followed by directional reactive ion etching to achieve site-selective exposure of core/shell wire tips and thus allow further structural modifications. Interestingly, this electrochemical deposition technique enables the synthesis of multilayered coaxial Ni/Au and Au/air shells on Si wire cores (Fig. 8e). Furthermore, conformal and uniform coating of Si micro/nanowires with different materials was unambiguously confirmed by elemental mapping images (Fig. 8f). Finally, the authors optimized the deposition conditions and showed that the described method can be generalized to different classes of materials (Fig. 8g). The described synthetic technique and fabrication technology can be useful for researchers working in the fields of plasmonics and metamaterials by providing unprecedented opportunities for realizing core/shell structures with nanoscale precision and controllability.

## Conclusions

In this review, we described a variety of novel plasmonic and metamaterial devices based on 1D subwavelength core/shell nanostructures, focusing on the rational design of core/shell NW structures for efficient manipulation of light–matter interaction. Importantly, we highlighted that the core/shell or core/multi-shell NW structures comprising plasmonic, dielectric, metasurface/metamaterials or two-dimensional materials exhibit unique optical properties with a variety of functions such as invisibility cloaking, super-scattering/super-absorption, enhanced luminescence and nonlinear optical activities, and deep subwavelength-scale optical waveguiding, which are rarely achievable in homogeneous single-wire structures. Experimental demonstrations of core/shell NW photonic devices such as invisible gold-covered Si NW sensors and enhanced luminescence in Si, CdS, and III–V NWs coupled to plasmonic nanocavities envision the further development of core/shell photonic devices. Furthermore, the recent advances in bottom-up synthesis combined with the top-down fabrication technologies for practical applications and the experimental realizations of functional 1D subwavelength core/shell nanostructure devices were briefly discussed. Thus, rational design and realization/controlled fabrication of subwavelength core/shell NW structures paves the way to the development of novel photonic devices free from the limitations of a homogenous single-wire structures.
